# Psychological benefits of outdoor physical activity in natural versus urban environments: A systematic review and meta‐analysis of experimental studies

**DOI:** 10.1111/aphw.12353

**Published:** 2022-03-08

**Authors:** Claire Wicks, Jo Barton, Sheina Orbell, Leanne Andrews

**Affiliations:** ^1^ School of Health and Social Care University of Essex Colchester UK; ^2^ School of Rehabilitation, Exercise and Sports Sciences University of Essex Colchester UK; ^3^ Department of Psychology University of Essex Colchester UK

**Keywords:** environment, green exercise, psychological health, physical activity

## Abstract

The impact of environmental context on the psychological benefits derived from physical activity has attracted research attention in recent years. Previous reviews have compared effects of indoor versus outdoor exercise. This review compares the effects of physical activity undertaken in outdoor green natural environments versus outdoor urban environments on psychological health outcomes in adult general populations. An electronic literature search identified 24 experimental studies meeting the inclusion criteria. Results were analysed via narrative synthesis (*n* = 24) and meta‐analysis (*n* = 9) of effect on six outcomes. Narrative synthesis found in favour of the natural environment for anxiety, anger/hostility, energy, affect and positive engagement. Post‐intervention effect sizes suggested duration and social context as potential moderators. The meta‐analyses revealed large or moderate effects in favour of the natural environment for anxiety, fatigue, positive affect and vigour, and a small effect for depression. Results were subject to high risk of bias and heterogeneity. Physical activity undertaken outdoors in natural environments is more beneficial for a range of psychological outcomes compared with urban environments. The various effect sizes evident in the meta‐analyses may be explained by differing mechanisms through which psychological gains are experienced during physical activity in nature.

## INTRODUCTION

In the past week, an estimated one in six people will have experienced a common mental health problem, such as depression or anxiety (McManus et al., 2016). Mental ill health is one of the most common causes of ill health and disability globally, affecting people of all ethnicities, ages and genders (GBD 2016 Disease and Injury Incidence and Prevalence Collaborators, 2018).

The burden of mental ill health is experienced by both the individual and wider society. Based on figures from 2010, the cost of mental ill health to the global economy was estimated to reach US$16 trillion by 2030 (Patel et al., 2018). Although various therapies and treatments are available, there is a disparity between the prevalence of mental ill health and funding for treatment. In 2012, 23% of all disease in the United Kingdom was attributed to mental ill health, yet it received only 13% of NHS health expenditure (The Centre for Economic Performance and Mental Health Policy Group, 2012). Vigo et al. (2019) report a disproportionate allocation of government funds for mental health in American countries, with the disease burden of mental ill health six times the proportion of funding. Estimates suggest that globally 70% of people suffering from mental ill health do not receive care from a healthcare professional (Henderson et al., 2013). In addition to inadequate funding, barriers including stigma (Van Voorhees et al., 2005), lack of perceived need for treatment (Mojtabai et al., 2011) and low outcome expectations of treatment (Bayer & Peay, 1997) are believed to prevent individuals from seeking treatment. Self‐management interventions that are readily available without diagnosis by a healthcare professional may provide individuals with tools that help maintain wellbeing and function and prevent exacerbation of symptoms. The present paper focuses on the role of physical activity in management of psychological wellbeing of the general population including those living with untreated symptoms of mental ill health (McManus et al., 2016).

Accumulated evidence suggests that physical activity may be as effective as psychological and pharmacological treatments for depression and anxiety (Cooney et al., 2013; Stubbs et al., 2017). Organisations including the UK National Institute of Clinical Excellence and World Health Organisation advocate physical activity as a strategy to help tackle mental ill health (National Institute for Health and Care Excellence, 2020; World Health Organisation, 2019), and recent years have seen the promulgation of so‐called ‘social prescribing’ in which people may be referred or self‐refer to non‐medical interventions including participation in physical activity.

### Physical activity contexts and psychological outcomes

The influence of the environmental setting in which physical activity takes place on psychological health outcomes has also received considerable research interest in recent years. Specifically, research has investigated whether physical activity while simultaneously exposed to elements of the natural environment, sometimes termed ‘green exercise’ (Pretty et al., 2005), might be more beneficial to psychological outcomes than physical activity in other types of manmade or synthetic environments such as indoor gyms.

Theories such as Psycho‐evolutionary Theory (Ulrich et al., 1991) and Attention Restoration Theory (Kaplan & Kaplan, 1989) propose potential mechanisms through which exposure to the natural environment can enhance health and wellbeing. Psycho‐evolutionary theory proposes that humans innately prefer natural environments that offer safety and resources required for survival. Being in such environments is associated with increased positive and reduced negative affective states. Attention Restoration Theory describes nature's ability to offer restoration of mental fatigue caused through depletion of directed attention. Humans use directed attention to focus on cognitively demanding tasks or situations and to maintain attention must constantly inhibit more interesting stimuli, resulting in mental fatigue. Spending time in the natural environment requires effortless or involuntary attention providing opportunity for the replenishment of fatigued directed attention.

Three previous reviews have considered the effect of environmental context on the psychological benefits of physical activity in adult populations. Two of these (Lahart et al., 2019; Thompson Coon et al., 2011) compared physical activity in natural versus indoor environments. Thompson Coon and colleagues reported greater feelings of revitalization and positive engagement, decreases in tension, confusion, anger and depression, and increased energy following physical activity in nature compared with indoor physical activity. Lahart et al. (2019) found that physical activity in nature (both actual and virtual) enhanced psychological outcomes for affective valence and enjoyment compared with indoor activity. However, the authors report equivocal findings for the effects on energy, calmness, tension, anger and depression. A third review (Bowler et al., 2010) compared exercise in natural vs ‘synthetic’ environments, with synthetic environments defined as indoor and ‘non‐green outdoor built environments’. The authors found in favour of the natural environment for anger, fatigue, tranquillity and sadness, with a marginally positive effect on energy.

Outdoor environments as a location for physical exercise confer many obvious benefits for self‐management of mental health and social prescribing. The outdoor environment can be accessed free of charge, unlike gym membership, and is available locally to all. However, because previous reviews have focused upon evidencing the benefits of outdoor versus indoor physical activity, it remains unknown whether features of the outdoor environment, namely whether it can be described as ‘green’ or ‘urban’ might impact upon the benefits of outdoor physical activity. In short, is it better to go for a walk in a town or in the countryside to improve your mental wellbeing? The aim of the present review is to address this question.

### Impact of gender on the effect of environmental context on outcomes

A secondary aim of the review was to investigate the possible moderating role of gender. Previous research has suggested that gender may influence the relationship between the environmental context of physical activity and psychological outcomes. Hassmen (1996) found that during outdoor running, women reported lower perceived exertion than expected based on their actual heart rate, whereas in men, perceived exertion was higher than expected. Barton and Pretty (2010) found that men experienced slightly larger improvements in mood following green exercise than women. Further, behaviour and attitudes towards natural environments has been found to differ between genders. Puett et al. (2014) found that men more likely to engage in physical activity outdoors, whereas Zelezny et al. (2000) report that women are more likely to engage in pro‐environmental behaviours and may have greater sense of being connected to the natural environment (Hughes et al., 2019). As connection with nature is a possible pathway between nature and health (Cervinka et al., 2012), this finding might indicate that women may experience greater psychological benefits from green exercise than men.

### Aims of the present study

This review extends previous research by addressing a clear gap in the literature. No previous review has compared the psychological health outcomes obtained from participating in physical activity in a natural versus an urban outdoor environment. However, the body of research addressing this topic has grown in recent years, making a synthesis of findings important and timely. As a free and readily available self‐management strategy, outdoor activity may be beneficial to the large population of people with common mental disorders who are not receiving treatment or support. Moreover, given an increased awareness of the potential negative consequences on psychological health of urban living (Gruebner et al., 2017), an understanding of the impact of environmental setting during physical activity might inform wellbeing and health policies and urban planning.

We conducted a systematic review and meta‐analysis of research comparing the effects of urban and natural outdoor exercise contexts. It was hypothesized based on the foregoing reviews that physical activity in nature will result in more favourable results for all psychological outcomes. We also sought to address gender as a moderator of the relationship of exercise context to outcomes. Based on previous research it was hypothesized that gender would moderate the relationship between physical activity in nature and enhanced psychological outcomes.

## METHOD

### Study registration

**FIGURE 1:**
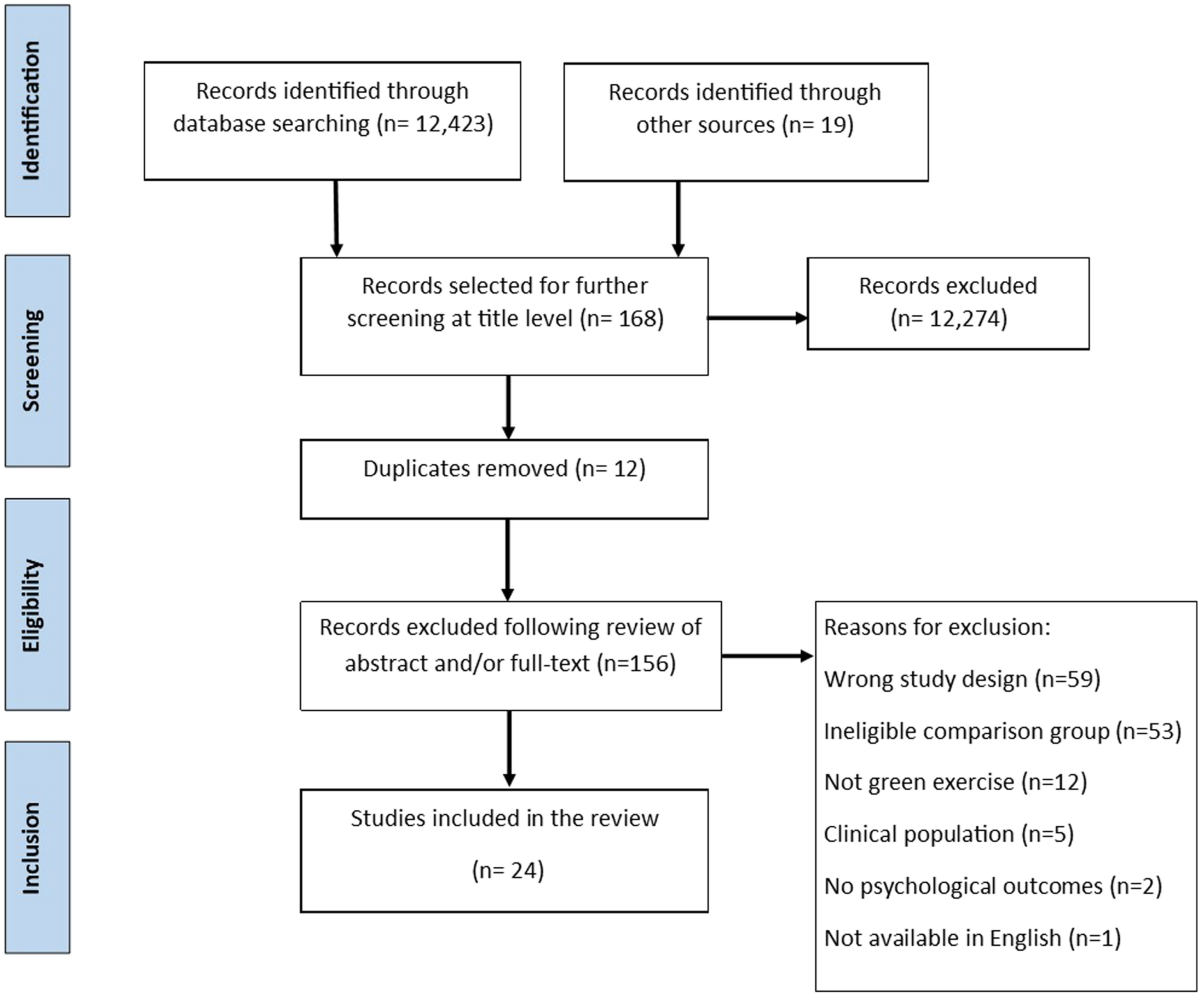
PRISMA flowchart of study identification and inclusion

The study protocol was registered at Prospero Journal in December 2019, Ref: CRD42019158162 (Wicks et al., 2019) and was conducted in accordance with the PRISMA guidelines (Moher et al., 2009). The PRISMA flowchart is shown in Figure 1.

### Search strategy and inclusion criteria

An electronic literature search was conducted using the following databases (from inception of the database to September 2019): Sport Discus, PsychInfo, PsychArticles, Greenfile, Medline (all via Ebsco host) and Web of Science. The title and abstract search included one of the following context keywords (Outdoor*,outside, forest, woodland, park*, ‘green space’, greenspace, ‘open space’, ‘green gym’, natur*, mountain*, garden*, allotment, wood*, horticulture*, wilderness, countryside, urban*, cit*, town*, borough*, built, built‐up ‘built up’, street*, suburban, metropolitan, industrial, developed, commercial, residential) together with one of the following physical activity keywords (AND ‘physical activity’, exerci*, fit*, sport*, hik*, walk*, jog*, run*, cycl*, climbing, kayak*, fishin, swimming, football, socce, surfing, volleyball, netball, hockey, rugby, recreation, conservation) and the following psychological outcomes (AND ‘mental health’, mood, well‐being, wellbeing, ‘well being’, self‐esteem, depress*, anxiety, stress, affect, emotion*, ‘quality of life’, psychological). The searches were also limited to adults (AND Men OR women OR male* OR female* or adult* NOT Child* OR adolescent OR youth).

### Eligibility screening and data extraction

Quantitative studies comparing physical activity outcomes in a natural and an urban environment were identified. Randomised controlled and non‐randomised studies, controlled pre and post studies, and cross‐over studies were eligible for inclusion. Studies were limited to those published in peer‐reviewed academic journals, in English language and where participants were adults (aged 18+ years) and from non‐clinical populations. Studies including participants with a known psychological, physical, or clinical diagnosis were excluded to avoid potential bias due to concomitant interventions or treatments, so as to ensure that outcomes can be attributed to green exercise. Eligible studies reported on psychological outcomes, broadly defined to include well‐being, self‐esteem, depression, anxiety, mood and stress. The protocol is available at https://www.crd.york.ac.uk/prospero/display_record.php?ID=CRD42019158162.

Potentially eligible studies were extracted into an Excel database. Papers were screened at abstract and full‐text level against the pre‐determined eligibility criteria. Any uncertainty around inclusion was discussed and resolved between C. W. and L. A. After screening, relevant data from each included article were extracted. Extracted data included participant characteristics, sample size, psychological outcomes reported, components of physical activity (type, duration, frequency and intensity), social context and descriptions of the natural and urban environments.

### Assessment of risk of bias

Risk of bias (RoB) was assessed for randomised controlled trials and randomised trials using the Revised Cochrane risk‐of‐bias tool for randomized trials (Sterne et al., 2019). Cross‐over studies were assessed for RoB using the tool adapted by Ding et al. (2015) which includes additional items specifically for crossover design studies (e.g. assessment of carry‐over effects between conditions). All studies were rated independently by CW and by JB. Inter‐rater reliability was assessed via mean agreement (82%) and Cohen's kappa (κ = .71). Discrepancies were resolved through discussion between C. W. and J. B.

### Narrative and meta‐analytic data synthesis

A narrative synthesis was undertaken following the protocol outlined by Popay et al. (2006). This involved identifying existing theories or models of green exercise and health that are supported by the findings, preliminary synthesis of findings, exploring relationships in the data and assessing the robustness of the synthesis. The synthesis of findings compares outcomes across studies, whereas effect sizes are employed as a common rubric to explore and interpret patterns in outcomes.

Meta‐analysis was conducted where at least two studies reported the same outcome and appropriate data for meta‐analysis was provided in the published paper or through contact with the authors. In total, nine of the 24 studies were included in the meta‐analyses. For crossover studies, the protocol for meta‐analysis outlined by Elbourne et al. (2002) was followed, which recommends using paired within participant data. Mean difference was used for meta‐analysis of studies using the same outcome measure to report a specific psychological construct. Standardised mean difference (SMD) was used where measures of a psychological construct differed between studies. Subgroup analysis was conducted using Borenstein and Higgins (2013)'s protocol. Meta‐analysis of data was conducted via random‐effects meta‐analysis in Review Manager, version 5.3, applying generic inverse variance outcomes for crossover studies and continuous outcomes for randomised trials. Heterogeneity of effects was investigated and reported using the *I* ^*2*^ statistic and interpreted as representing considerable heterogeneity where values of 75% or higher were returned (Higgins et al., 2003). The chi‐squared statistic was also considered with *p* values of ≤.10 indicating heterogeneity of the intervention effects. The lower criterion value was used due to the reduced power of chi‐square where a small number of studies are included in meta‐analysis (Deeks et al., 2021). The pooled mean or SMD for each psychological outcome included in the meta‐analysis is reported with 95% confidence intervals. SMD is interpreted with effects of 0.2 as small, 0.5 moderate and >0.8 large (Cohen, 1998). It was not appropriate to assess publication bias via funnel plots as the tests would be underpowered to distinguish chance from real asymmetry due to the small number of studies in each meta‐analysis (Page et al., 2021).

## RESULTS

### Study design characteristics

The searches identified 24 unique studies for inclusion (Figure 1). Studies were crossover trials (*n* = 18), randomised trials (*n* = 4) or randomised controlled trials (*n* = 2). Twenty‐two studies were based on a single bout of physical activity with a duration ranging from 15 to 60 min. Studies were conducted in Japan (*n* = 6), USA (*n* = 6), United Kingdom (*n* = 3), Sweden (*n* = 2), Finland (*n* = 2), China (*n* = 2), with one study each in Canada, Taiwan and Denmark (Table 1).

**TABLE 1:** Characteristics of included studies

Study & country	Study design	Sample size	Age (mean or range) ± 1SD	Gender	Type of activity	Social context	Duration/distance	Intensity	Natural environment	Urban environment	
Bodin and Hartig (2003)^a^ Sweden	XOVR	12	26–46	50% female	Running	Alone	60 min (14 km)	Relaxed pace	Maintained path through pine‐birch forest, open fields and lakeside.	Medium‐density residential and commercial area.	
Bratman et al. (2015) America	RT	70	24.1 (SD not published)	53% female	Walking	Unspecified	50 min	Unspecified ‘similar levels of exertion’	Paved path through grassland with scattered shrubs and oak trees.	Busy street with multiple lanes of traffic. Some bushes and trees along sidewalk.	
Butryn and Furst (2003) America	XOVR	30	31 ± 10.5	All female	Running	Alone	4 miles	Self‐rated comfortable pace	Highly vegetated route with occasional views of a creek	Industrial area near downtown with heavy vehicular and pedestrian traffic.	
DeBrito et al. (2019) America	XOVR	24	49.3 ± 6.7	83% female	Walking	Alone	50 min	Self‐paced	Unpaved trail including secluded areas surrounded by large trees, grassland areas and a pond.	Route with medium density traffic in a residential development area.	
Geniole et al. (2016) Canada	XOVR	31	24.61 ± 3.9	Males	Walking	Alone	15 ± 3 min	Moderate comfortable pace	Regenerated landfill area, resembling green park, with visible methane release pipes	Business and commercial district with grass along most of the sidewalks	
Gidlow et al. (2016) UK	XOVR	38	40.9 ± 17.6	39% female	Walking	Alone^b^	30 min	Self‐paced	Country park within the city	Quiet residential streets with low levels of traffic compared with UK average.	
Han (2017) Taiwan	RT	116	20.85 ± 1.1	55% female	Walking & jogging	Unspecified	15 min	Low (walking) or moderate (jogging)	Natural road lined with vegetation	Built road with buildings lining both sides	
Hartig et al. (1991) America	RCT	102	20 (SD not published)	50% female	Walking	With a guide	40 min	Intensity not specified	Park with stream and associated riparian habitat, some ornamental vegetation and chaparral flora	Centre of a large, well‐kept diverse urban area, with commercial and residential buildings	
Hartig et al. (2003) America	RT	112	20.87 ± 3.7	50% female	Walking	With an assistant	50 min	Low intensity	Well graded dirt track through 4000‐acre vegetation and nature reserve	Medium‐density professional office and retail development with visible landscaped areas.	
Hassan et al. (2018) China	XOVR	60	19.6 ± 1.4	50% female	Walking	Alone	15 min	Intensity not specified	Track through a well‐managed bamboo forest area	Urban area with many traditional buildings	
Johansson et al. (2011) Sweden	XOVR	20	20–29	50% female	Walking	Alone & with a friend	40 min	Brisk pace	Path through park passing open fields, gold course, river and landscaped area	City streets with moderate levels of traffic, low rise buildings of varying architecture and colour.	
Kinnafick and Thøgersen‐Ntoumani (2014) UK	XOVR	30	25.86 ± 11.5	56% female	Walking	Alone	15 min	Pace set by guide, but not specified	Park primarily made up of green space with small areas of woodland and a pond	Busy and built up commercially dominant area.	
Lyu et al. (2019) China	RT	120	19–24	50% female	Walking	Groups of 30	15 min	Moderate intensity	2× bamboo forests, 1 bamboo park	Centre of downtown area	
Ojala et al. (2019) Finland	XOVR	88	48.31 ± 8.6	100% female	Walking	Groups of up to 4	30 min	Slow walking, paced by guide	1 × 1000 ha urban woodland, with diverse animal and plant life; 1× constructed urban park, with water and recreational features.	City Centre with heavy traffic, museums, shopping and traffic Centre.	
Perkins et al. (2011) America	RT	26	19–24	73% female	Walking	Groups of 2–4	20 min	Moderate pace	Wooded trail, partially snow covered	Residential area and parking lot	
Roe et al. (2011) Scotland	XOVR	11^c^	46 (SD not published)	64% female	Walking	Groups of approx. 10	60 min	Intensity not specified	70 ha park with woodland, wilderness and parkland	Stirling town Centre with some greenery and historic interest
Song et al. (2013) Japan	XOVR	13	22.5 ± 3.1	100% male	Walking	Alone	15 min	Intensity not specified	Urban park; trees had either lost their leaves or turned red or yellow.	City area around the urban park
Song et al. (2014) Japan	XOVR	17	21.2 ± 1.7	100% males	Walking	Alone	15 min	Intensity not specified	Urban park; trees had light green leaves, and the azaleas were in full bloom	City area around the urban park
Song et al. (2015) Japan	XOVR	23	22.3 ± 1.2	100% males	Walking	Alone	15 min	Intensity not specified	Urban park with hardwood trees and a large pond	City area around the park, including a residential area
Song et al. (2018) Japan	XOVR	624	27.1 ± 1.6	100% males	Walking	Alone	15 min	Intensity not specified	52× different safe and well‐maintained forest areas	52× urban areas, either downtown or near a railway station
Song et al. (2019) Japan	XOVR	60	21 ± 1.3	100% female	Walking	Alone	15 min	Participant's normal walking pace	6× different well‐maintained forest areas	6× urban areas, either downtown or near a railway station
Stigsdotter et al. (2017) Demark	XOVR	51	20–36	100% female	Walking	Groups of 4–5	15 min	Intensity not specified	Health forest (2 ha, diverse vegetation and rich nature)	Historical downtown area; varied historical and architectonical qualities, with few natural elements.
Takayama et al. (2014) Japan	XOVR	45	Mean age by group: 21.2 ± 0.8 20.8 ± 1.5 21.4 ± 1.3 21.1 ± 1.4	100% male	Walking	Alone	15 min	Intensity not specified	2× forests consisting of mainly Japanese cedar, and 2× forests consisting of Japanese and Sawtooth oak.	4× downtown areas along major traffic roads or around the main station in each district
Tyrväinen et al. (2014) Finland	XOVR	95	47.64 ± 8.68 (based on final sample of 77)	92% female (of the 77)	Walking	Group of up to 4	30 min	Slow walking speed	1 × 20 ha. Urban park, including a water feature, flower beds and grass lawns. 1 × 1000 ha forest area, with mixed and conifer forests.	City Centre next to the main city street, with a few trees visible.

Abbreviations: RCT, randomised controlled trial; RT, randomised trial; XOVR, crossover design.

^a^ Participants ran twice in each condition.

^b^ Researcher followed one pace behind and asked for rating of exertion at 5 min intervals.

^c^ Only 11 participants from the good mental health group are considered.

### Participant characteristics

A total of 1800 participants were included across the 24 included studies. Samples were described as university or college students (*n* = 16), university staff and students (*n* = 1), adults (*n* = 5) or regular runners (*n* = 2). Based on the eligibility criteria of the individual studies, participants were healthy and without physical or mental health conditions. Participant mean age ranged from 19 to 49.3 years (Table 1).

### Experimental conditions: Context and type of physical activity

All studies compared a natural and urban condition (Table 1). Natural environments included forest, woodland, grasslands, regenerated landfill, nature reserve, urban park and bamboo forests. Urban environments included commercial districts, historical downtown areas, central city areas or residential streets. The level of detail used to describe environmental conditions varied, with some studies providing photographs in addition to written descriptions, for example, Stigsdotter et al. (2017). Participants undertook walking (*n* = 22), jogging (*n* = 1) and running (*n* = 2). Han (2017) compared both walking and jogging in each environment. Duration ranged from 15 min to 1 h. Intensity of physical activity was specified in 14 studies, with six studies measuring intensity objectively using an actigraph and velocity obtained via a Nike+ running app (Han, 2017), average heart rate (Kinnafick & Thøgersen‐Ntoumani, 2014) or heart rate variability (Gidlow et al., 2016; Song et al., 2013; Song et al., 2014; Song et al., 2019). The remaining 10 studies did not specify intensity.

### Assessment of risk of bias for randomised trials and crossover studies

**FIGURE 2:**
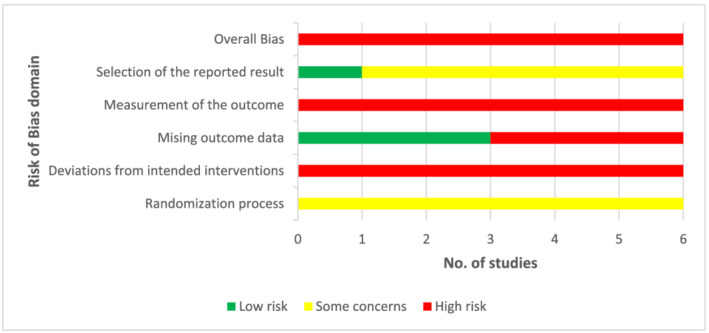
Assessment of risk of bias for randomised trials

Risk of bias assessments for randomised trials are summarised in Figure 2. Randomisation procedures were not fully reported and three of the six included randomised studies did not clearly report the number of participants whose data were included in outcome analyses. Although blinding participants to their condition was not possible, studies where participants were familiarised with the environmental conditions prior to the experiment may have resulted in biased reporting and were therefore rated more highly for RoB. The overall RoB rating for all six randomised studies was high; however, this rating may be inflated due to inadequate reporting.

**FIGURE 3:**
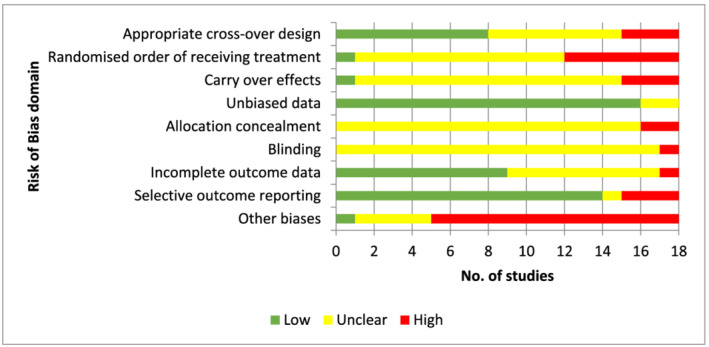
Assessment of risk of bias for crossover studies

Inadequate reporting also affected assessment of RoB for crossover studies (Figure 3). Eleven of the 18 crossover studies randomly allocated participants, but only Gidlow et al. (2016) reported adequate details of participant randomisation. Carry‐over effects were not reported in any of the studies. Studies were rated as high risk (*n* = 3) where both conditions were undertaken within 24 h, or unclear (*n* = 14) where the washout period was at least 1 week. de Brito et al. (2019) reported a 2‐week washout period and was rated as low RoB. Most crossover studies reported unbiased data; however, two studies were unclear. Bodin and Hartig (2003) presented data collapsed by condition, whereas Roe and Aspinall (2011) collected data using nine validated variables and produced a new factor structure, reporting results for the four variables derived from the factor structure. Allocation of concealment was not reported in any crossover studies. Two studies were rated as high RoB where the published procedures gave no details of any attempt to conceal the allocation of participants (Roe & Aspinall, 2011; Stigsdotter et al., 2017). All other studies were rated as unclear (*n* = 16). It was indeterminate whether any blinding of participants or study personnel was attempted in 16 of the 18 crossover studies. Selective outcome reporting was rated as high for three crossover studies where outcome data were incomplete. Thirteen crossover studies also rated as high for biases including small sample sizes and lack of information around participant recruitment.

### Narrative synthesis results

Narrative synthesis was undertaken for all 24 studies shown in Table 1, and results are summarised here by outcome variable. The effect sizes obtained in each study are detailed in Table S1.

#### Anxiety

Sixteen studies reported outcomes for anxiety. Eleven of these reported statistically significant decreases in anxiety following physical activity in a natural environment compared with an urban environment. Lyu et al. (2019) reported statistically significant decreases following a combination of seated viewing and walking in two bamboo forest conditions compared with a downtown area, but not between the bamboo park and downtown conditions. Three studies reported non‐significant improvements in favour of the natural environment (Bodin & Hartig, 2003; Han, 2017; Stigsdotter et al., 2017).

#### Depression

Four out of 12 studies reported statistically significant decreases in depression scores in favour of the natural environment. As for anxiety, Lyu et al. (2019) reported depression significantly decreased in the two bamboo forest conditions compared with the downtown condition, but not in the bamboo park condition. Four studies reported non‐significantly lower depression scores in the natural environment. However, effect sizes indicated a moderate effect in favour of the natural environment, *d* = −0.58 and *d* = −0.47 for Song et al. (2014) and Song et al. (2015), respectively, but may have been underpowered to detect significant changes. Perkins (2011) reported a significant pre‐post decrease regardless of environmental setting (all *p*'s < .05). Three studies found no significant difference between conditions (Han, 2017; Hartig et al., 1991; Song et al., 2013).

#### Anger and hostility

Half (6/12) of the studies considering anger reported statistically significant decreases in the natural environment. Hartig et al. (2003) suggest that increased mental fatigue negated the beneficial effects of the natural environment. Perkins (2011) reported significant reductions in anger (*p* < .01) regardless of condition. Three possibly underpowered studies (Song et al., 2013; Song et al., 2014; Stigsdotter et al., 2017) reported non‐significant reductions in anger in the natural condition but had moderate to large effect sizes. One study reported no significant difference between conditions (Han, 2017).

#### General affect, positive emotions and engagement

Twenty‐four outcomes relating to general affect, positive emotions or engagement were reported across 10 individual studies. Positive affect improved in favour of the natural environment in six of eight studies. Two studies measuring affective valence and happiness also reported in favour of the natural environment. Hartig et al. (2003) found that participants who undertook an attentional task reported lower happiness scores in the natural environment, compared with a non‐task group. The opposite effect was found in the urban environment. Negative affect significantly reduced in favour of the natural condition in 4/5 studies. Tyrväinen et al. (2014) reported a significant reduction when comparing the forest and urban conditions, with a non‐significant reduction between the park and urban conditions. Two studies reported on positive engagement and reported scores increased in both conditions. For attentiveness, one study reported decreases in both conditions and a second no difference between conditions. A single study measured arousal and found no difference between conditions.

#### Energy

Energy includes revitalisation, vitality, fatigue, vigour and lack of exhaustion. All 12 studies found physical activity in nature was more beneficial for increased energy or reduced fatigue. Song et al. (2013) reported significant decreases in fatigue in both conditions. In Kinnafick and Thøgersen‐Ntoumani (2014) energy increased following a 15‐min walk in both conditions but decreased in the urban condition following 15‐min of sitting.

For vigour 7/10 studies found statistically significant increases in favour of the natural environment. Stigsdotter et al. (2017) found a non‐significant improvement (*d* = 0.37). Butryn and Furst (2003) reported significant improvements in revitalisation in both conditions. Johansson et al. (2011) reported revitalisation significantly increased only when walking alone in the natural environment or while walking with a friend in the urban condition. Tyrväinen et al. (2014) found that subjective vitality increased in the forest and park conditions and decreased in the city condition. Ojala et al. (2019) reported low urban‐orientated participants had significantly higher vitality scores in the forest compared with park conditions and decreased scores in the urban condition. In high urban‐orientated participants, vitality was higher in all conditions.

#### Tranquillity and calmness

Four crossover trials investigated measures of tranquillity or calmness. One study reported a statistically significant increase in tranquillity following the park condition, but not in the urban condition (Butryn & Furst, 2003). Two studies reported increased scores regardless of the environmental condition (Bodin & Hartig, 2003; Johansson et al., 2011). Kinnafick and Thøgersen‐Ntoumani (2014) reported no statistically significant effects of the environment on calmness, when weather variables were included as covariates in analyses.

### Summary of narrative synthesis

Although there are some inconsistencies across outcomes, this analysis revealed results generally supporting our hypothesis. The majority of tests showed greater benefits following green exercise for anxiety, anger/hostility, energy, general affect and engagement, whereas four out of 10 tests found in favour of the natural environment for depression and one in four for tranquillity. Where studies did not find in favour of the natural environment, the results often indicated either favourable changes in both conditions or no changes in either condition.

### Patterns identified in narrative synthesis

Post‐intervention between groups effect sizes displayed in Table S1 were used to identify potential relationships between green exercise and psychological health. Although gender was hypothesised to influence outcomes, no effect was evident in the narrative analysis when comparing effect sizes between genders. However, a difference by gender for anxiety was noted by Bodin and Hartig (2003). Two potential moderators were identified and are discussed here: duration of green exercise and social context of green exercise.

#### Duration of green exercise

For anxiety, 15‐min of walking in the natural environment resulted in large (*d* ≥ −0.80) (Hassan et al., 2018; Song et al., 2013; Song et al., 2015; Song et al., 2019) or moderately‐large effect sizes (*d* = −0.59 to *d* = −0.64) (Kinnafick & Thøgersen‐Ntoumani, 2014; Song et al., 2014). In contrast, for durations of 50‐min small (*d* = −0.06) and moderate (*d* = −0.31) effects were obtained by Bratman et al. (2015) and de Brito et al. (2019) respectively. For negative affect, 15‐min of walking resulted in a large effect (*d* = −0.64) (Kinnafick & Thøgersen‐Ntoumani, 2014), which reduced to a small effect at 30‐min (*d* = −0.18) (Tyrväinen et al., 2014) and a trivial effect at 50‐min (*d* = −0.08) (Bratman et al., 2015). For both walking and running, effect sizes for tranquillity/calmness decreased as duration increased. A large effect was found after a 15‐min walk (*d* = 0.84) (Kinnafick & Thøgersen‐Ntoumani, 2014), which decreased to a small to moderate effect after 40‐min (*d* = 0.38) (Johansson et al., 2011). In women, a moderate effect size following a 4‐mile run (*d* = 0.42) (Butryn & Furst, 2003) decreased after a 14 km (approximately 8.7 miles) run (*d* = 0.28). The difference in effect sizes between walking and running might indicate tranquillity is affected by mode of physical activity or intensity.

#### Social context

The effect sizes reported in four studies suggest that positive affect increased to a greater extent when green exercise was undertaken in the presence of others. An effect size of *d* = 0.26 was found where female participants walked alone in a forest area (de Brito et al., 2019) compared with effect sizes ranging between *d* = 1.43 and *d* = 0.61 (Hartig et al., 2003) where participants walked in groups or had a researcher in close proximity (i.e. guiding the walk). Further, Johansson et al. (2011) reported more favourable scores for tranquillity following a 40‐min walk with a friend (alone: *d* = 0.38, with friend: *d* = 0.60), anxiety and depression (alone: *d* = 0.02, with friend: *d* = −0.05), anger (alone: *d* = −0.10, with friend: *d* = −0.25) and physical exhaustion (alone: *d* = 0.05, with friend: *d* = 0.08).

### Meta‐analysis results

As previously noted, data from nine studies contributed to meta‐analyses of six different outcomes (Table 2). Forest plots for each outcome are shown in Figure S1a–f. The results of all six meta‐analyses indicated effects in favour of the natural environment; however, the effect size and heterogeneity differed between outcomes. Large or moderate effect sizes were obtained for anxiety, fatigue and positive affect (*d* = −6.59, −1.98 and 0.59, respectively), but considerable heterogeneity was also evident. For vigour, a large effect (*d* = 3.28) in favour of the natural environment had low heterogeneity. A moderate effect was found for anger, with low heterogeneity (*d* = −0.57). The meta‐analysis for depression revealed a small effect (*d* = 0.34) in favour of the natural environment, but with considerable heterogeneity.

**TABLE 2:** Results of meta‐analyses comparing psychological outcomes of outdoor physical activity in natural and urban environments

Outcome	No. of participants (studies)	Statistical method	Effect estimate [95% CI]	*I* ^2^	*χ* ^2^ (*df*)
Anxiety	720 (7)	Std. mean difference (IV, random, 95% CI)	−6.59 [−10.04, −3.13]*	91%	66.98 (*df* = 6)**
Depression	697 (5)	Mean difference (IV, random, 95% CI)	−0.34 [−0.62, −0.05]*	74%	15.12 (*df* = 4)**
Anger/hostility	697 (5)	Mean difference (IV, random, 95% CI)	−0.57 [−0.79, −0.35]*	30%	5.71 (*df* = 4)
Fatigue	697 (5)	Mean difference (IV, random, 95% CI)	−1.98 [−2.77, −1.19]*	79%	19.18 (*df* = 4)**
Vigour	697 (5)	Mean difference (IV, random, 95% CI)	3.28 [2.84, 3.71]*	15%	4.73 (*df* = 4)
Positive affect	115 (2)	Std. mean difference (continuous, random, 95% CI)	0.59 [0.21, 0.98]*	92%	12.43 (*df* = 1)**

*Note*: *I* ^2^ indicates the level of heterogeneity in the meta‐analysis; ≥70 = considerable heterogeneity.

* Statistically significant outcome in favour of the natural environment (*p* < .05).

** Chi‐squared test indicates significant heterogeneity (*p* ≤ .10).

### Sub‐group analysis: Moderation by gender

Sufficient data were available to conduct sub‐group analysis by gender for the anxiety outcome. Five studies had single sex samples (men: *n* = 4; women: *n* = 1), and results were provided independently by gender for one study (Table 1). The results revealed no significant difference by gender (SMD women = −6.49, SMD men = −4.49, *p* = .64), with similar effects in favour of the natural environment for men and women (Figure S1g). The *I* ^*2*^ statistics for the male (*I* ^*2*^ = 86%) and female (*I* ^*2*^ = 95%) subgroups indicated considerable heterogeneity in both sub‐groups, suggesting that there is another factor, not dependent on gender, causing study heterogeneity. Insufficient data were available to conduct sub‐group analyses by duration and social context.

## DISCUSSION

The aim of this review was to synthesize evidence from experimental studies comparing the effect of physical activity in natural versus urban environments on psychological outcomes. A systematic literature review identified 24 studies eligible for inclusion, 16 of these published since 2014. All 24 studies were included in the narrative synthesis, and data were available to include nine studies in meta‐analyses. Psychological outcomes of physical exercise considered across studies were anxiety, depression, anger/hostility, positive affect, positive engagement, energy/vigour/fatigue, tranquillity and calmness.

The narrative synthesis found largely in favour of the natural environment for anxiety, anger/hostility, affect and positive engagement and energy. Exploration of patterns by effect size suggested that duration of green exercise and social context may be moderators of effect. The results of the meta‐analyses were consistent with the results of the narrative synthesis showing significant differences between natural and urban contexts in improvements in anxiety, depression, anger/hostility, fatigue, vigour and positive affect following physical activity. Effect size was large for anxiety, fatigue and vigour. Small or moderate effect sizes were obtained for other outcomes. The hypothesised moderator analysis by gender revealed no significant difference in the effect of green versus urban exercise.

Although previous reviews have provided evidence for the benefits of outdoor versus indoor exercise, the present study is the first to synthesize recently accumulated evidence to evaluate the impact of the type of outdoor environment in which physical activity is pursued. Evidence suggests that outdoor context may be crucial to consider in order to maximize the psychological benefits of physical activity.

### Effect of environment on anxiety and depression

The results of the meta‐analysis showed that whereas physical activity in natural environments was associated with significant reductions in anxiety and depression compared with urban environments, the effect size obtained for anxiety was very large, whereas the effect size for depression was small. The narrative synthesis also found consistently in favour of the natural environment for anxiety, whereas results for depression were inconsistent, similar to the findings of Lahart et al. (2019). It is plausible that natural and urban outdoor environments have different impacts on anxiety and depression. For example, natural environments may offer respite from anxiety by providing both a physical and mental space that is free from everyday stressors. In contrast anxiety may be not be alleviated by busy urban environments. Physical activity undertaken alone in natural versus urban environments may confer fewer benefits for symptoms of depression. Natural environments may enhance sense of loneliness or increase opportunities for rumination (Berman et al., 2012). Urban environments may offer a sense of connection or identity (Bornioli et al., 2018).

The studies reviewed primarily evaluated a single bout of physical activity within each environment. The large effect size obtained for anxiety may reflect greater sensitivity of anxiety to the restorative effects of the natural environment so that symptoms change rapidly and changes were detectible in the study designs utilised. Depression may require longer or repeated exposure to generate the same effect sizes as seen in anxiety. At present, the cumulative effects of multiple sessions on both anxiety and depression are unknown. The impact of exposure may vary by unmeasured variables or interactions with type of outcome (benefit to anxiety versus depression) and social context. Furthermore, the analysis presented here is based only on pre and immediately post intervention data. As such, it tells us little of the longevity of any beneficial effects experienced. It could be that although smaller in size, green exercise had a more prolonged effect on depression. These are important questions for future research.

### Effect of environment on other psychological outcomes

The meta‐analysis revealed large effect sizes for both fatigue and vigour in favour of the natural environment. Physical activity in a green environment resulted in reduced fatigue and increased vigour compared with exercise in an urban environment. These findings augment previous observations that outdoor exercise reduces fatigue and increases vigour relative to the effects of indoor exercise (Bowler et al., 2010; Thompson Coon et al., 2011). It would appear that outdoor exercise conducted in a green environment confers particular benefits in restoring fatigue, consistent with attention restoration theory. In addition, a moderate effect size was obtained in the meta‐analysis for positive affect, and reduced negative affect was observed in the narrative synthesis. These findings are consistent with psycho‐evolutionary theory (Ulrich et al., 1991). However, Brymer et al. (2021) suggests that existing theories may not capture the full range of possible experiences in nature.

### Potential moderators of effect

The relative impact of green versus urban exercise by gender was tested via sub‐group analysis in the meta‐analysis. Data were available for one outcome, anxiety, and no difference by gender was observed. Due to the small number of studies available for analysis, the question of whether gender moderates the effect of the environment on psychological outcomes remains unanswered. Using the effect sizes calculated as part of the narrative synthesis, duration and social context were observed as potential moderators. However, insufficient data were available to conduct statistical moderator analysis of these variables.

The narrative review suggested that shorter exercise sessions were associated with larger effect sizes for both anxiety and negative affect. Short durations were also found to be most beneficial for mood and self‐esteem in a multi‐study review (Barton & Pretty, 2010). Ulrich (1983) suggests that initial responses to environments may be pre‐cognitive and occur quickly based on global and generalised affects (e.g. like or dislike). That the effect of duration was not evident for all outcomes highlights the varying response of psychological outcomes to environmental setting.

Social context was also derived as a potential moderator from the narrative review. Although social interaction was not encouraged in any of the studies, having others with a shared purpose (participating in or conducting the study) nearby may have provided feelings of social support or cohesion. The presence of others might also offer greater feelings of safety or reassurance in natural areas that could otherwise feel secluded or unsafe.

The suggested relationships between psychological outcomes and duration of green exercise and social context are tentative and require further exploration. Evidence suggests that complex interactions are in operation such that it matters who is being physically active, with whom and for how long, as well as in what context. These factors may also have differential benefit for anxiety outcomes that may be more sensitive to environmental surroundings and depression outcomes that rely upon stimulation and invigoration or social contact to obtain benefit.

### Robustness of narrative synthesis

The quality assessment of all studies using a validated tool highlighted similar areas of concern across all studies. These included carryover and order effects not assessed in crossover trials and lack of blinding of study personnel across all studies. Due to the homogeneity in level of risk of bias across studies, all studies were given equal consideration in the review. The methodological limitations of the studies synthesised limits the conclusions that can be drawn. Although some potential moderators between outcomes were identified, these were based on between groups post‐intervention effect sizes, which were not available for all studies. As such, these findings should be considered preliminary and used to inform future research.

### Strengths and limitations

The present review has many strengths including the pre‐registration of the study protocol, use of PRISMA guidelines and a validated tool to assess risk of bias. Combining narrative and meta‐analysis methodologies and the consensus between findings strengthens confidence in findings. Further, the included studies were limited to those where participants were fully immersed in actual natural and urban environments unlike previous reviews which included comparisons with virtual environments (e.g. Lahart et al., 2019).

The reliance on student samples in the majority of included studies limits generalisability to other populations. Further, studies eligible for inclusion focused only on the effectiveness of walking and jogging/running in natural and urban environments. Alternative activities such as cycling might be considered. Several of the studies included in the meta‐analyses for anxiety, depression, fatigue and vigour were from the same primary author. Although this may be advantageous due to increased consistency in study design and protocols, they also share some methodological weaknesses which may have resulted in favourable outcomes for the natural environment. As in the narrative synthesis, the outcomes of the assessment of RoB influences the reliability of the meta‐analyses, as do the high *I* ^*2*^ scores and issues around availability of data.

### Future directions

We recommend replication of this review in the future as more original studies become available, to both extend the meta‐analysis and to investigate the potential moderators identified here. To this end, we recommend researchers design studies that provide evidence of varying durations and social context and also address the limitations identified here including longer term follow up that will permit evaluation of effect over time. This will support the development of a theoretical framework which explains the mechanisms by which undertaking physical activity in green environments impacts psychological outcomes. This will inform understanding of for whom and in which contexts this activity is beneficial. Future reviews may also wish to include clinical samples to identify whether the effect of physical activity in natural and urban environments differs between clinical and general populations. We also call to researchers to make data readily available to facilitate future meta‐analyses.

### Conclusion

Physical activity in nature may be more beneficial for a range of psychological outcomes, than physical activity undertaken in urban environments. Benefits are particularly evident for reduction in feelings of anxiety or fatigue, with somewhat weaker evidence for other outcomes including depression. The variation in the results of the meta‐analyses may be explained by differing mechanisms through which psychological gains are experienced during physical activity in nature. This review provides the first synthesis of evidence to show that outdoor activity in nature improves mental health more than outdoor activity in urban environments. These findings have important implications for urban planning and green social prescribing for mental health.

## Supporting information


**Figure S1.** a. Anxiety; b. Vigour; c. Depression; d. Positive Affect; e. Anger/hostility; f. Fatigue; g. Sub‐group analysis Forest Plot

**Table S1:** Between groups post intervention effect sizes

## Data Availability

Data sharing is not applicable to this article as no new data were created or analysed in this study.

## References

[aphw12353-bib-0001] Barton, J. , & Pretty, J. (2010). What is the best dose of nature and green exercise for improving mental health? A multi‐study analysis. Environmental Science & Technology, 44(10), 3947–3955. 10.1021/es903183r 20337470

[aphw12353-bib-0002] Bayer, J. , & Peay, M. (1997). Predicting intentions to seek help from professional mental health services. The Australian and New Zealand Journal of Psychiatry, 31(4), 504–513. 10.3109/00048679709065072 9272260

[aphw12353-bib-0003] Berman, M. G. , Kross, E. , Krpan, K. M. , Askren, M. K. , Burson, A. , Deldin, P. J. , Kaplan, S. , Sherdell, L. , Gotlib, I. H. , & Jonides, J. (2012). Interacting with nature improves cognition and affect for individuals with depression. Journal of Affective Disorders, 140(3), 300–305. 10.1016/j.jad.2012.03.012 22464936 PMC3393816

[aphw12353-bib-0004] Bodin, M. , & Hartig, T. (2003). Does the outdoor environment matter for psychological restoration gained through running? Psychology of Sport and Exercise, 4(2), 141–153. 10.1016/s1469-0292(01)00038-3

[aphw12353-bib-0005] Borenstein, M. , & Higgins, J. P. T. (2013). Meta‐analysis and subgroups. Prevention Science, 14(2), 134–143. 10.1007/s11121-013-0377-7 23479191

[aphw12353-bib-0006] Bornioli, A. , Parkhurst, G. , & Morgan, P. L. (2018). The psychological wellbeing benefits of place engagement during walking in urban environments: A qualitative photo‐elicitation study. Health & Place, 53, 228–236. 10.1016/j.healthplace.2018.08.018 30195155

[aphw12353-bib-0007] Bowler, D. E. , Buyung‐Ali, L. M. , Knight, T. M. , & Pullin, A. S. (2010). A systematic review of evidence for the added benefits to health of exposure to natural environments. BMC Public Health, 10(1), 456. 10.1186/1471-2458-10-456 20684754 PMC2924288

[aphw12353-bib-0008] Bratman, G. N. , Daily, G. C. , Levy, B. J. , & Gross, J. J. (2015). The benefits of nature experience: Improved affect and cognition. Landscape and Urban Planning, 138, 41–50. 10.1016/j.landurbplan.2015.02.005

[aphw12353-bib-0009] Brymer, E. , Crabtree, J. , & King, R. (2021). Exploring perceptions of how nature recreation benefits mental wellbeing: A qualitative enquiry. Annals of Leisure Research, 24(3), 394–413. 10.1080/11745398.2020.1778494

[aphw12353-bib-0010] Butryn, T. M. , & Furst, D. M. (2003). The effects of park and urban settings on the moods and cognitive strategies of female runners. Journal of Sport Behavior, 26(4), 335–355. Retrieved from <Go to WoS>://WOS:000369368900011

[aphw12353-bib-0011] Cervinka, R. , Röderer, K. , & Hefler, E. (2012). Are nature lovers happy? On various indicators of well‐being and connectedness with nature. Journal of Health Psychology, 17(3), 379–388. 10.1177/1359105311416873 21859800

[aphw12353-bib-0012] Cohen, J. (1998). Statistical power analysis for the behavioral sciences, statistical power analysis for the behavioral sciences. Laurence Erlbaum Associates.

[aphw12353-bib-0013] Cooney, G. M. , Dwan, K. , Greig, C. A. , Lawlor, D. A. , Rimer, J. , Waugh, F. , McMurdo, M. , & Mead, G. E. (2013). Exercise for depression. Cochrane Database of Systematic Reviews, 9. 10.1002/14651858.CD004366.pub6 PMC9721454 24026850

[aphw12353-bib-0014] de Brito, J. N. , Pope, Z. C. , Mitchell, N. R. , Schneider, I. E. , Larson, J. M. , Horton, T. H. , & Pereira, M. A. (2019). Changes in psychological and cognitive outcomes after green versus suburban walking: A pilot crossover study. International Journal of Environmental Research and Public Health, 16(16). 10.3390/ijerph16162894 PMC6719990 31412602

[aphw12353-bib-0015] Deeks, J. J. , Higgins, J. P. T. , & Altman, D. G. (Eds.) (2021). Chapter 10: Analysing data and undertaking meta‐analyses. In J. P. T. Higgins , J. Thomas , J. Chandler , M. Cumpston , T. Li , M. J. Page , & Welch, V. A. (Eds.). Cochrane handbook for systematic reviews of interventions version 6.2 (updated February 2021). Retrieved from www.training.cochrane.org/handbook

[aphw12353-bib-0016] Ding, H. , Hu, G. L. , Zheng, X. Y. , Chen, Q. , Threapleton, D. E. , & Zhou, Z. H. (2015). The method quality of cross‐over studies involved in Cochrane systematic reviews. PLoS ONE, 10(4), e0120519. 10.1371/journal.pone.0120519 25867772 PMC4395015

[aphw12353-bib-0017] Elbourne, D. R. , Altman, D. G. , Higgins, J. P. , Curtin, F. , Worthington, H. V. , & Vail, A. (2002). Meta‐analyses involving crossover trials: Methodological issues. International Journal of Epidemiology, 31(1), 140–149. 10.1093/ije/31.1.140 11914310

[aphw12353-bib-0018] GBD 2016 Disease and Injury Incidence and Prevalence Collaborators . (2018). Global, regional, and national incidence, prevalence, and years lived with disability for 354 diseases and injuries for 195 countries and territories, 1990–2017: A systematic analysis for the global burden of Disease study 2017. The Lancet, 392(10159), 1989–1858. 10.1016/S0140-6736(18)32279-7 PMC6227754 30496104

[aphw12353-bib-0019] Gidlow, C. J. , Jones, M. V. , Hurst, G. , Masterson, D. , Clark‐Carter, D. , Tarvainen, M. P. , Smith, G. , & Nieuwenhuijsen, M. (2016). Where to put your best foot forward: Psycho‐physiological responses to walking in natural and urban environments. Journal of Environmental Psychology, 45, 22–29. 10.1016/j.jenvp.2015.11.003

[aphw12353-bib-0062] Gruebner, O. , Rapp, M. A. , Adli, M. , Kluge, U. , Galea, S. , & Heinz, A. (2017). Cities and Mental Health. Deutsches Ärzteblatt International, 114(8), 121–127. 28302261 10.3238/arztebl.2017.0121 PMC5374256

[aphw12353-bib-0020] Han, K. T. (2017). The effect of nature and physical activity on emotions and attention while engaging in green exercise. Urban Forestry & Urban Greening, 24, 5–13. 10.1016/j.ufug.2017.03.012

[aphw12353-bib-0021] Hartig, T. , Evans, G. W. , Jamner, L. D. , Davis, D. S. , & Gärling, T. (2003). Tracking restoration in natural and urban field settings. Journal of Environmental Psychology, 23(2), 109–123. 10.1016/S0272-4944(02)00109-3

[aphw12353-bib-0022] Hartig, T. , Mang, M. , & Evans, G. W. (1991). Restorative effects of natural environment experiences. Environment and Behavior, 23(1), 3–26. 10.1177/0013916591231001

[aphw12353-bib-0023] Hassan, A. , Tao, J. , Li, G. , Jiang, M. Y. , Aii, L. , Jiang, Z. H. , Liu, Z. F. , & Chen, Q. B. (2018). Effects of walking in bamboo Forest and City environments on brainwave activity in young adults. Evidence‐Based Complementary and Alternative Medicine., 2018, 1–9. 10.1155/2018/9653857 PMC5896408 29785198

[aphw12353-bib-0024] Hassmen, P. (1996). Environmental effects on ratings of perceived exertion in males and females. Journal of Sport Behavior, 19(3).

[aphw12353-bib-0025] Henderson, C. , Evans‐Lacko, S. , & Thornicroft, G. (2013). Mental illness stigma, help seeking, and public health programs. America Journal of Public Health, 103(5), 777–780. 10.2105/ajph.2012.301056 PMC3698814 23488489

[aphw12353-bib-0026] Higgins, J. P. T. , Thompson, S. G. , Deeks, J. J. , & Altman, D. G. (2003). Measuring inconsistency in meta‐analyses. BMJ, 327(7414), 557–560. 10.1136/bmj.327.7414.557 12958120 PMC192859

[aphw12353-bib-0027] Hughes, J. , Rogerson, M. , Barton, J. , & Bragg, R. (2019). Age and connection to nature—When is engagement critical? Frontiers in Ecology & the Environment., 17(5), 265–269. 10.1002/fee.2035

[aphw12353-bib-0028] Johansson, M. , Hartig, T. , & Staats, H. (2011). Psychological benefits of walking: Moderation by company and outdoor environment. Applied Psychology: Health and Well‐Being, 3(3), 261–280. 10.1111/j.1758-0854.2011.01051.x

[aphw12353-bib-0029] Kaplan, R. , & Kaplan, S. (1989). The experience of nature: A psychological perspective. Cambridge University Press.

[aphw12353-bib-0030] Kinnafick, F.‐E. , & Thøgersen‐Ntoumani, C. (2014). The effect of the physical environment and levels of activity on affective states. Journal of Environmental Psychology, 38, 241–251. 10.1016/j.jenvp.2014.02.007

[aphw12353-bib-0031] Lahart, I. , Darcy, P. , Gidlow, C. , & Calogiuri, G. (2019). The effects of green exercise on physical and mental wellbeing: A systematic review. International Journal of Environmental Research and Public Health, 16(8). 10.3390/ijerph16081352 PMC6518264 30991724

[aphw12353-bib-0032] Lyu, B. Y. , Zeng, C. C. , Deng, S. Y. , Liu, S. L. , Jiang, M. Y. , Li, N. , Wei, L. Q. , Yu, Y. , & Chen, Q. B. (2019). Bamboo forest therapy contributes to the regulation of psychological responses. Journal of Forest Research, 24(1), 61–70. 10.1080/13416979.2018.1538492

[aphw12353-bib-0033] McManus, S. , Bebbington, P. , Jenkins, R. , & Brugha, T. (2016). Mental Health and Wellbeing in England: Adult Psychiatric Morbidity Survey, 2014. Retrieved from. https://files.digital.nhs.uk/pdf/q/3/mental_health_and_wellbeing_in_england_full_report.pdf

[aphw12353-bib-0034] Moher, D. , Liberati, A. , Tetzlaff, J. , & Altman, D. G. (2009). Preferred reporting items for systematic reviews and meta‐analyses: The PRISMA statement. BMJ, 339, b2535. 10.1136/bmj.b2535 19622551 PMC2714657

[aphw12353-bib-0035] Mojtabai, R. , Olfson, M. , Sampson, N. , Jin, R. , Druss, B. , Wang, P. , Wells, K. , Pincus, H. , & Kessler, R. C. (2011). Barriers to mental health treatment: Results from the National Comorbidity Survey Replication. Psychological Medicine, 41(8), 1751–1761. 10.1017/S0033291710002291 21134315 PMC3128692

[aphw12353-bib-0036] National Institute for Health and Care Excellence . (2020). The NICE guideline on the treatment and management of depression in adults (2nd ed.). Retrieved from https://www.nice.org.uk/guidance/cg90/evidence/full-guidline-pdf-4840934509

[aphw12353-bib-0037] Ojala, A. , Korpela, K. , Tyrvainen, L. , Tiittanen, P. , & Lanki, T. (2019). Restorative effects of urban green environments and the role of urban‐nature orientedness and noise sensitivity: A field experiment. Health & Place, 55, 59–70. 10.1016/j.healthplace.2018.11.004 30502229

[aphw12353-bib-0038] Page, M. J. , Higgins, J. P. T. , & Sterne, J. A. C. (2021). Chapter 13: Assessing risk of bias due to missing results in a synthesis. In J. P. T. T. Higgins , J. Chandler , M. Cumpston , T. Li , M. J. Page , & V. A. Welch (Eds.), Cochrane handbook for systematic reviews of interventions version (6.2 ed.).

[aphw12353-bib-0039] Patel, V. , Saxena, S. , Lund, C. , Thornicroft, G. , Baingana, F. , Bolton, P. , … UnÜtzer, J. (2018). The Lancet Commission on Global Mental Health and Sustainable Development. The Lancet, 392(10157), 1553–1598. 10.1016/S0140-6736(18)31612-X 30314863

[aphw12353-bib-0040] Perkins, S. (2011). Walking in a natural winter setting to relieve attention fatigue: A pilot study. Psychology, 02, 777–780. 10.4236/psych.2011.28119

[aphw12353-bib-0041] Popay, J. , Roberts, H. , Sowden, A. , Petticrew, M. , Arai, L. , Rodgers, M. , Britten, N. , Roen, K. , & Duffy, S. (2006). Guidance on the conduct of narrative synthesis in systematic reviews: A product from the ESRC methods Programme.

[aphw12353-bib-0042] Pretty, J. , Peacock, J. , Sellens, M. , & Griffin, M. (2005). The mental and physical health outcomes of green exercise. International Journal of Environmental Health Research, 15(5), 319–337. 10.1080/09603120500155963 16416750

[aphw12353-bib-0043] Puett, R. , Teas, J. , España‐Romero, V. , Artero, E. G. , Lee, D.‐c., Baruth, M. , Sui, X. , Montresor‐López, J. , & Blair, S. N. (2014). Physical activity: Does environment make a difference for tension, stress, emotional outlook, and perceptions of health status? Journal of Physical Activity & Health, 11(8), 1503–1511. 10.1123/jpah.2012-0375 24733227

[aphw12353-bib-0044] Roe, J. , & Aspinall, P. (2011). The restorative benefits of walking in urban and rural settings in adults with good and poor mental health. Health & Place, 17(1), 103–113. 10.1016/j.healthplace.2010.09.003 21094074

[aphw12353-bib-0045] Song, C. , Ikei, H. , Igarashi, M. , Miwa, M. , Takagaki, M. , & Miyazaki, Y. (2014). Physiological and psychological responses of young males during spring‐time walks in urban parks. Journal of Physiological Anthropology, 33, 8. 10.1186/1880-6805-33-8 24887352 PMC4041337

[aphw12353-bib-0046] Song, C. , Joung, D. , Ikei, H. , Igarashi, M. , Aga, M. , Park, B.‐J. , Miwa, M. , Takagaki, M. , & Miyazaki, Y. (2013). Physiological and psychological effects of walking on young males in urban parks in winter. Journal of Physiological Anthropology, 32, 18. 10.1186/1880-6805-32-18 24168929 PMC3817995

[aphw12353-bib-0047] Song, C. R. , Ikei, H. , Igarashi, M. , Takagaki, M. , & Miyazaki, Y. (2015). Physiological and psychological effects of a walk in urban parks in fall. International Journal of Environmental Research and Public Health, 12(11), 14216–14228. 10.3390/ijerph121114216 26569271 PMC4661642

[aphw12353-bib-0048] Song, C. R. , Ikei, H. , Kagawa, T. , & Miyazaki, Y. (2019). Physiological and psychological effects of viewing forests on young women. Forests, 10(8). 10.3390/f10080635 24858505

[aphw12353-bib-0049] Sterne, J. , Savović, J. , Page, M. , Elbers, R. , Blencowe, N. , Boutron, I. , Cates, C. , Cheng, H.‐Y. , Corbett, M. , Eldridge, S. , Emberson, J. , Hernán, M. , Hopewell, S. , Hróbjartsson, A. , Junqueira, D. , Juni, P. , Kirkham, J. , Lasserson, T. , Li, T. , & Higgins, J. (2019). RoB 2: A revised tool for assessing risk of bias in randomised trials. BMJ, 366, l4898. 10.1136/bmj.l4898 31462531

[aphw12353-bib-0050] Stigsdotter, U. K. , Corazon, S. S. , Sidenius, U. , Kristiansen, J. , & Grahn, P. (2017). It is not all bad for the grey city—A crossover study on physiological and psychological restoration in a forest and an urban environment. Health & Place, 46, 145–154. 10.1016/j.healthplace.2017.05.007 28528275

[aphw12353-bib-0051] Stubbs, B. , Koyanagi, A. , Hallgren, M. , Firth, J. , Richards, J. , Schuch, F. , Rosenbaum, S. , Mugisha, J. , Veronese, N. , Lahti, J. , & Vancampfort, D. (2017). Physical activity and anxiety: A perspective from the world health survey. Journal of Affective Disorders, 208, 545–552. 10.1016/j.jad.2016.10.028 27802893

[aphw12353-bib-0052] The Centre for Economic Performance and Mental Health Policy Group . (2012). How mental illness loses out in the NHS. Retrieved from http://cep.lse.ac.uk/_new/research/mentalhealth/default.asp

[aphw12353-bib-0053] Thompson Coon, J. , Boddy, K. , Stein, K. , Whear, R. , Barton, J. , & Depledge, M. (2011). Does participating in physical activity in outdoor natural environments have a greater effect on physical and mental wellbeing than physical activity indoors? A systematic review. Environmental Science & Technology, 45(5), 1761–1772. 10.1021/es102947t 21291246

[aphw12353-bib-0054] Tyrväinen, L. , Ojala, A. , Korpela, K. , Lanki, T. , Tsunetsugu, Y. , & Kagawa, T. (2014). The influence of urban green environments on stress relief measures: A field experiment. Journal of Environmental Psychology, 38, 1–9. 10.1016/j.jenvp.2013.12.005

[aphw12353-bib-0063] Ulrich, R. S. (1983). Aesthetic and affective response to natural environment. In I. Altman & J. F. Wohlwill (Eds.), Behavior and the Natural Environment. Human Behavior and Environment (Advances in Theory and Research) (Vol. 6), Springer.

[aphw12353-bib-0055] Ulrich, R. S. , Simons, R. F. , Losito, B. D. , Fiorito, E. , Miles, M. A. , & Zelson, M. (1991). Stress recovery during exposure to natural and urban environments. Journal of Environmental Psychology, 11(3), 201–230. 10.1016/s0272-4944(05)80184-7

[aphw12353-bib-0056] Van Voorhees, B. W. F. , Houston, J. , Thomas, K. , Cooper, L. A. , Wang, N. , & Ford, D. E. (2005). Beliefs and attitudes associated with the intention to not accept the diagnosis of depression among young adults. Annals of Family Medicine, 3(1), 38–45. 10.1370/afm.273 15671189 PMC1466793

[aphw12353-bib-0057] Vigo, D. V. , Kestel, D. , Pendakur, K. , Thornicroft, G. , & Atun, R. (2019). Disease burden and government spending on mental, neurological, and substance use disorders, and self‐harm: Cross‐sectional, ecological study of health system response in the Americas. The Lancet Public Health, 4(2), e89–e96. 10.1016/S2468-2667(18)30203-2 30446416

[aphw12353-bib-0058] Wicks, C. , Andrews, L. , Barton, J. , & Orbell, S. (2019). Does environmental setting influence the psychological outcomes gained through outdoor physical activity in adults? A systematic review and meta‐analysis. Retrieved from https://www.crd.york.ac.uk/prospero/display_record.php?

[aphw12353-bib-0059] World Health Organisation . (2019). Motion for your mind: Physical activity for mental health promotion, protection and care. Retrieved from https://www.euro.who.int/__data/assets/pdf_file/0018/403182/WHO-Motion-for-your-mind-ENG.pdf?ua=1

[aphw12353-bib-0060] Zelezny, L. C. , Chua, P.‐P. , & Aldrich, C. (2000). Elaborating on gender differences in environmentalism. Journal of Social Issues, 56(3), 443–457. 10.1111/0022-4537.00177

