# Supplementary material for: Psychological benefits of outdoor physical activity in natural versus urban environments: A systematic review and meta‐analysis of experimental studies
Source: Appl Psychol Health Well Being. 2022 Mar 8;14(3):1037–61. doi: 10.1111/aphw.12353 (PMC9544808; doi:10.1111/aphw.12353)
Supplement: Supplementary file 1 — Figure S1. a. Anxiety; b. Vigour; c. Depression; d. Positive Affect; e. Anger/hostility; f. Fatigue; g. Sub‐group analysis Forest Plot [file APHW-14-1037-s001.docx]

**Supplementary materials 2: Forest Plots**

**Figure S1a. Anxiety Figure S1b.Vigour**


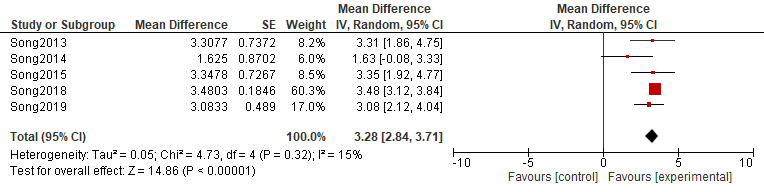
**
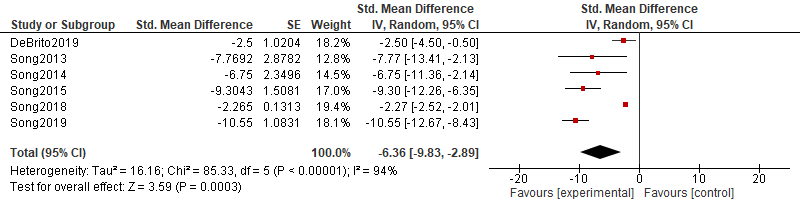
**

**Figure S1c. Depression Figure S1d. Positive Affect**


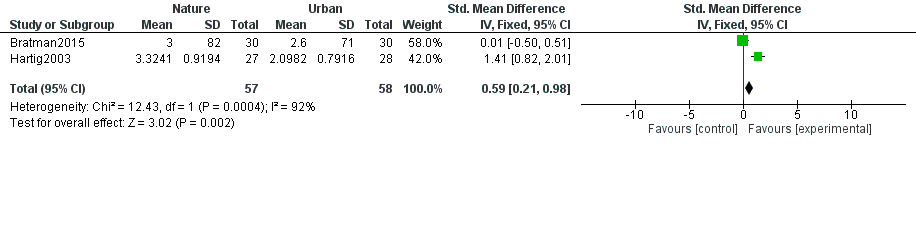
**
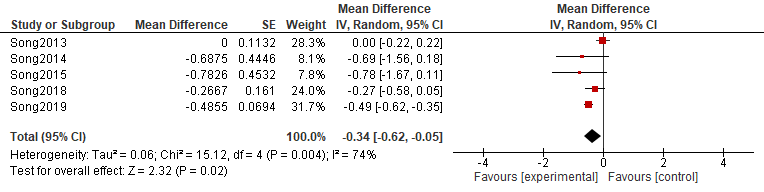
**

**Figure S1e. Anger/hostility Figure S1f. Fatigue**


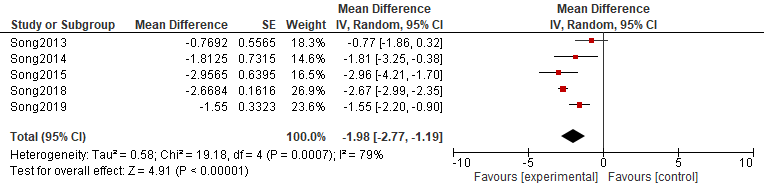
**
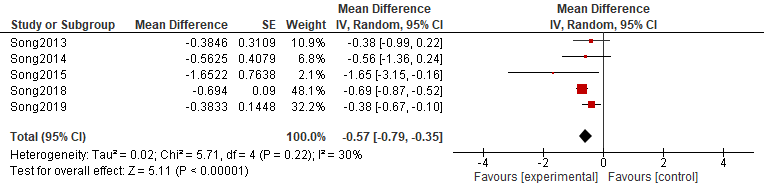
**

**Supplementary Materials Figure S1g. Sub-group analysis Forest Plot**


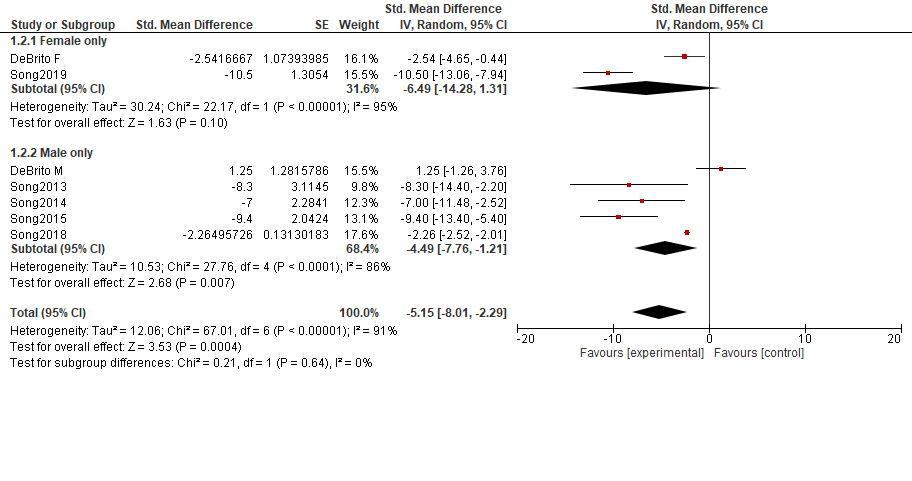


Females

Males

All
