# Supplementary material for: Psychological benefits of outdoor physical activity in natural versus urban environments: A systematic review and meta‐analysis of experimental studies
Source: Appl Psychol Health Well Being. 2022 Mar 8;14(3):1037–61. doi: 10.1111/aphw.12353 (PMC9544808; doi:10.1111/aphw.12353)
Supplement: Supplementary file 2 — Table S1: Between groups post intervention effect sizes [file APHW-14-1037-s002.pdf]

Supplementary materials Table S1: Between groups post intervention effect sizes.

Author	Duration	Social context	Natural setting	Anxiety	Tension / anxiety / nervousness	Anxiety & Depression	Depression / sadness	Anger / Hostility	Fatigue / Physical Exhaustion	Vigour	Revitilisation	Energy	Vitality	Total Mood Disturbance	Positive Engagement	Attentiveness	Happiness
Bodin (men)	14km (approx 1	Alone	Park			d = -0.70 [8]		d = -1.22 [8]			d = 0.21 [4]						
Bodin (women)	14km	Alone	Park			d = 0.04 [8]		d = 0.11 [8]			d = 0.16 [4]						
Bratman	50 min	Unspecified	Park	d = -0.06 [1]													
Butryn	4 miles	Alone	Park								d = 0.08 [4]			d = 0.10 [3]	d = 0.09 [4]		
DeBrito	50 min	Alone	Forest	d = -0.31 [1]													
Gidlow	30 min	Alone (researcher tra	Park											0.05 [5]			
Han [a]	15 min	Not specified	Path through natural area		d = -3.67 [3]				d = -3.36 [3]								
Hartig 2003 (no task group)	50 min	With assistant	Park inc. wooded area				d =-0.08 [13]	d = -1.05 [13]								d = 0.03 [13]	d= 0.77 [14]
Hartig 2003 (task group)	50 min	With assistant	Park inc. wooded area				d = 0.17 [13]	d = -0.13 [13]								d = 0.29 [13]	d = -0.34 [14]
Hassan	15 min	Alone	Bamboo forest	d = -0.80 [1]													
Johansson (alone)	40 min	Alone	Park			d = 0.02 [8]		d = -0.10 [8]	d = 0.05 [4]		d = 0.36 [4]				d = 0.35 [4]		
Johansson (with friend)	40 min	With friend	Park			d = -0.05 [8]		d = -0.25	d = 0.08 [4]		d = 0.26 [5]				d = 0.35 [5]		
Kinnafick	15 min	Alone	Park inc. wooded area		d = -0.64 [9]				d = 0.13 [9]			d = 0.34 [9]					
Ojala	30 min	Groups of up to 4	Forest										d = 0.68 [10]				
Ojala	30 min	Groups of up to 4	Park										d = 0.62 [10]				
Roe	60 min	Group of approx 10	Park		d = -0.27 [15]							d = -0.20 [15]		d = 0.79 [15]			
Song 2019 x 6 sites	15 min	Alone	Forest x 6 sites	d = -1.47 [1]	d = -0.76 [3]		d = -0.18 [3]	d = -0.50 [3]	d = -0.83 [3]	d = 0.98 [3]							
Song 2018 x 52 sites	15 min	Alone	Forest		d = -0.78 [3]		d = -0.25 [3]	d = -0.37 [3]	d = -0.72 [3]	d = 0.85 [3]							
Song 2015	15 min	Alone	urban park	d = -1.40 [1]	d = -1.00 [3]		d = -0.47 [3]	d = -0.67 [3]	d = -0.97 [3]	d = 0.72 [3]							
Song 2014	15 min	Alone	urban park	d = -1.05 [1]	d = -0.59 [3]		d = -0.58 [3]	d = -0.55 [3]	d = -0.49 [3]	d = 0.51 [3]							
Song 2013	15 min	Alone	urban park	d= -1.10 [1]	d = -0.94 [3]		d = 0 [3]	d = -0.39 [3]	d = -0.37 [3]	d = 0.77 [3]							
Stigsdotter	15 min	Groups of 4-5	urban park		d = -1.11 [3]		d = -0.11 [3]	d = -0.47 [3]	d = -0.16 [3]	d = 0.37 [3]				d = -0.53 [3]			
Tyrväinen	30 min	Groups of up to 4	Park										d = 0.64 [10]				
Tyrväinen	30 min	Groups of up to 4	Forest										d = 0.70 [10]				

1 = State Trait Anxiety Index; 2= Postive and Negative Affect Schedule; 3 = Profile of Mood States; 4 = Exercise Feeling Inventory; 5 = BRUMS; 6= Feeling Scale;
7 = Affect Grid; 8 =Negative Mood Scale; 9 = Activation-Deactivation Adjective Check List; 10 = Subjective Vitality Scale; 11 = Rosenberg Self-Esteem Scale;
12 = Mood Adjective Checklist; 13 = Inventory of Personal Reactions (ZIPERS); 14 = Overall Happiness Scale; 15= Mood Adjective Checklist, 16 = Felt Arousal Scale;
17 = Perceived Restorativeness Scale, 18 = Restoration Outcome Scale.
a = Includes walking and jogging conditions.

Large effect in favour of natural environment (d= >0.80)
Moderate effect in favour of natural environment (d=0.50)
Small effect in favour of natural environment (d =0.20)
No difference between enviroments
Small effect in favour of urban environment (d=0.20)
Moderate effect in favour of the urban environment (d=0.50)

Means and standard deviations (SD) were unavailable in Geniole (2016), Hartig (1991), Perkins (2011), Lyu (2019) & Tyrväinen (2014). Han (2017) only reports means and SD for significant outcomes.
Means and SDs pubished within the individual studies are available upon request from the first author.

Author	Duration	Social context	Natural setting	Tranquility / calmness	Positive Affect	Negative Affect	Affective valence (pleasure/displeasure)	Arousal	Restoration (extent from PRS)	Fear (arousal)	Confusion	Self-esteem
Bodin (men)	14km (approx 1 hour)	Alone	Park	d = 0.24 [4]								
Bodin (women)	14km	Alone	Park	d = 0.28 [4]								
Bratman	50 min	Unspecified	Park		d=0.52 [2]	d= -0.08 [2]						
Butryn	4 miles	Alone	Park	d = 0.42 [4]								
DeBrito	50 min	Alone	Forest		d = 0.27 [2]	d = -0.36 [2]						
Gidlow	30 min	Alone (researcher traile	Park									
Han [a]	15 min	Not specified	Path through natural area									
Hartig 2003 (no task group)	50 min	With assistant	Park inc. wooded area		d = 1.43 [13]					d = 0.26 [13]		
Hartig 2003 (task group)	50 min	With assistant	Park inc. wooded area		d = 0.60 [13]					d = 0.17 [13]		
Hassan	15 min	Alone	Bamboo forest									
Johansson (alone)	40 min	Alone	Park	d = 0.38 [4]								
Johansson (with friend)	40 min	With friend	Park	d = 0.60 [5]								
Kinnafick	15 min	Alone	Park inc. wooded area	d = 0.84 [9]	d = 0.55 [2]	d = -0.64 [2]	d = 1.02 [6]	d =-0.37 [16]				
Ojala	30 min	Groups of up to 4	Forest						d = 1.01 [18]			
Ojala	30 min	Groups of up to 4	Park						d = 0.83 [18]			
Roe	60 min	Group of approx 10	Park									d =-0.24 [11]
Song 2019 x 6 sites	15 min	Alone	Forest x 6 sites								d = -0.45 [3]	
Song 2018 x 52 sites	15 min	Alone	Forest								d = -0.41 [3]	
Song 2015	15 min	Alone	urban park								d = -0.86 [3]	
Song 2014	15 min	Alone	urban park								d = -0.40 [3]	
Song 2013	15 min	Alone	urban park								d =-0.43 [3]	
Stigsdotter	15 min	Groups of 4-5	urban park								d = -0.55 [3]	
Tyrväinen	30 min	Groups of up to 4	Park		d = 0.67 [2]	d = -0.18 [2]			d = 0.87 [18]			
Tyrväinen	30 min	Groups of up to 4	Forest		d = 0.72 [2]	d = -0.48 [2]			d = 1.07 [18]			

1 = State Trait Anxiety Index; 2= Postive and Negative Affect Schedule; 3 = Profile of Mood States; 4 = Exercise Feeling Inventory; 5 = BRUMS; 6= Feeling Scale;
7 = Affect Grid; 8 =Negative Mood Scale; 9 = Activation-Deactivation Adjective Check List; 10 = Subjective Vitality Scale; 11 = Rosenberg Self-Esteem Scale;
12 = Mood Adjective Checklist; 13 = Inventory of Personal Reactions (ZIPERS); 14 = Overall Happiness Scale; 15= Mood Adjective Checklist, 16 = Felt Arousal Scale;
17 = Perceived Restorativeness Scale, 18 = Restoration Outcome Scale.
a = Including walking and jogging conditions.

Large effect in favour of natural environment (d= >0.80)
Moderate effect in favour of natural environment (d=0.50)
Small effect in favour of natural environment (d =0.20)
No difference between enviroments
Small effect in favour of urban environment (d=0.20)
Moderate effect in favour of the urban environment (d=0.50)
